# Longitudinal changes in bone mineral density among patients with hyperthyroidism: A prospective cohort study

**DOI:** 10.6026/973206300213055

**Published:** 2025-09-30

**Authors:** Preeti Nigotia, Archana Patel, Poorva Parihar, Abhinav Sharma

**Affiliations:** 1 Department of General Medicine, SRVS medical college, Shivpuri, Madhya Pradesh, India; 2 Department of Pharmacology, Smt. B. K. Shah Medical Institute and Research Centre, Sumandeep Vidyapeeth, Waghodia, Vadodara, Gujarat, India; 3 Department of General Medicine, NSC Government Medical College Khandwa, Madhya Pradesh, India; 4 Department of Orthopaedic, Government Medical College, Satna, Madhya Pradesh, India

**Keywords:** Hyperthyroidism, bone mineral density, osteopenia, osteoporosis, DEXA scan, prospective cohort, thyrotoxicosis, bone health

## Abstract

Bone mineral density (BMD) changes over one year in 124 newly diagnosed hyperthyroid patients were analysed. Hence, dual-energy X-ray absorptiometry (DEXA) scans was performed at baseline and after 12 months of treatment. Significant reductions in BMD were noted at the lumbar spine and femoral neck at baseline, with partial recovery following restoration of euthyroid state. Older age and prolonged thyrotoxicosis were associated with less BMD improvement. Thus, we show the need for early screening and long-term bone health monitoring in hyperthyroid patients.

## Background:

Hyperthyroidism increases bone turnover, which, in turn, may lead to bone loss from the spine and hip [1]. Hyperthyroidism, particularly in its overt form, has been well-documented as a secondary cause of low bone mineral density (BMD), leading to increased risk of osteopenia, osteoporosis and fragility fractures [2]. Low bone mineral density (BMD) and increased bone turnover are common features of untreated hyperthyroidism in adult patients. The effect of treatment on BMD is still controversial [3]. Thyroid hormones influence bone turnover by stimulating both osteoblast and osteoclast activity, but the resorptive effect predominates, resulting in net bone loss, especially in cortical-rich skeletal sites like the femoral neck and lumbar spine [4]. Patients with untreated or chronic hyperthyroidism often experience accelerated bone remodeling, calcium mobilization and decreased bone strength [5]. While previous studies have demonstrated the detrimental impact of hyperthyroidism on skeletal integrity, data on longitudinal BMD changes following definitive treatment such as anti-thyroid therapy, radioiodine, or surgery-remain limited [6]. The extent to which BMD can recover after achieve a euthyroid state and the factors influencing this recovery, are crucial for guiding long-term management of bone health in hyperthyroid patients [7]. This prospective cohort study was designed to evaluate the pattern of BMD changes over a 12-month period among newly diagnosed hyperthyroid patients undergoing standard treatment. Therefore, it is of interest to identify the degree of bone recovery following restoration of thyroid function and assess predictors of persistent low BMD despite biochemical improvement.

## Materials and Methods:

This prospective cohort study was conducted at a tertiary endocrine center between January 2022 and December 2023. A total of 124 adult patients (age ≥18 years) with newly diagnosed overt hyperthyroidism were enrolled. Diagnosis was based on suppressed serum TSH (<0.01 mIU/L) with elevated free T4 and/or free T3 levels. Patients with known osteoporosis, chronic steroid use, renal or hepatic disease, parathyroid disorders, or prior thyroid treatment were excluded. At baseline, all patients underwent detailed clinical evaluation, laboratory tests (TSH, free T4, free T3, calcium, ALP, vitamin D) and bone mineral density assessment using dual-energy X-ray absorptiometry (DEXA) at the lumbar spine (L1-L4) and femoral neck. Patients were started on anti-thyroid treatment (carbimazole or propylthiouracil) with individualized dosing. Follow-up assessments were conducted quarterly and repeat DEXA scans were performed at 12 months post-treatment initiation. Patients achieving euthyroid state (TSH 0.5-4.5 mIU/L) were included in the final analysis. The primary outcome was the change in BMD (g/cm^2^) at lumbar spine and femoral neck after one year. Secondary outcomes included prevalence of osteopenia and osteoporosis and identification of factors (age, gender, baseline T4 levels and duration of thyrotoxicosis) associated with BMD improvement. Data were analyzed using paired t-tests, chi-square tests and multivariate regression using SPSS v26. A p-value <0.05 was considered statistically significant.

## Results:

Of the 124 patients enrolled, 112 completed the 12-month follow-up and were included in the final analysis. At baseline, 64.3% had osteopenia and 17.9% had osteoporosis at one or more skeletal sites. After 12 months of treatment and achievement of euthyroid state, significant improvement in BMD was observed, particularly at the lumbar spine. However, patients with older age and longer symptom duration before diagnosis showed less BMD recovery. Table 1 (see PDF) shows the majority of patients were female and between 31-50 years of age. Table 2 (see PDF) shows all patients had elevated T4 and suppressed TSH at baseline. Table 3 (see PDF) shows at baseline, osteopenia was more common than osteoporosis, with the lumbar spine being more affected. Table 4 (see PDF) shows Mean BMD at both sites improved significantly after 12 months of treatment. Table 5 (see PDF) shows the greatest BMD gain occurred among younger patients (<50 years). Table 6 (see PDF) shows Patients with a shorter duration of thyrotoxic symptoms had greater BMD recovery. Table 7 (see PDF) shows Vitamin D insufficiency was common and associated with lower BMD improvement. Table 8 shows Males showed slightly greater BMD recovery than females at the femoral neck. Table 9 (see PDF) shows the proportion of patients with osteoporosis declined after 12 months of treatment. Table 10 (see PDF) shows Multivariate regression showed that age >50, symptom duration >12 months and vitamin D deficiency were independent predictors of poor BMD recovery.

## Discussion:

This prospective cohort study demonstrates that hyperthyroidism significantly impairs bone mineral density, with a majority of newly diagnosed patients presenting with osteopenia or osteoporosis. Importantly, restoration of euthyroid status over 12 months resulted in measurable improvement in BMD, particularly at the lumbar spine and femoral neck [8]. These findings affirm the skeletal reversibility of thyroid hormone excess when treatment is initiated early and maintained consistently. Female predominance and a high prevalence of BMD loss at diagnosis mirror patterns seen in existing literature, reflecting both hormonal vulnerability and delayed detection [9]. Younger patients and those with a shorter duration of thyrotoxicosis showed greater BMD recovery, reinforcing the importance of early intervention. In contrast, older individuals and those with prolonged untreated disease had persistently lower BMD, likely due to more extensive and irreversible micro architectural damage [10]. Vitamin D deficiency was highly prevalent and significantly associated with reduced BMD gain. This highlights the synergistic role of thyroid hormones and vitamin D in bone remodeling. Adequate correction of vitamin D levels may therefore enhance BMD restoration, especially in populations with high baseline deficiency rates [11]. Interestingly, the improvement in BMD was modest but statistically significant, emphasizing the need for prolonged monitoring beyond biochemical remission [12]. Although DEXA scores improved, 12.5% of patients still met criteria for osteoporosis at 1 year, suggesting that the skeletal effects of hyperthyroidism may persist beyond hormonal normalization [13]. These patients may benefit from adjunctive interventions such as bisphosphonates, nutritional support and physical activity guidance [14]. This study underscores the importance of baseline and serial BMD assessments in patients with hyperthyroidism, especially in older adults and postmenopausal women. Proactive bone health strategies must be integrated into thyroid management protocols to minimize fracture risk and long-term morbidity.

## Conclusion:

Hyperthyroidism significantly impairs bone mineral density, but partial recovery is achievable within 12 months of treatment. Early diagnosis, correction of vitamin D deficiency and close skeletal monitoring are essential, particularly in older patients and those with delayed presentation. Integrating bone health evaluation into routine hyperthyroid care can reduce the burden of osteoporosis and improve long-term outcomes.
